# A novel splice site mutation in *AP1S2* gene for X‐linked mental retardation in a Chinese pedigree and literature review

**DOI:** 10.1002/brb3.1221

**Published:** 2019-02-04

**Authors:** Liang Huo, Ziteng Teng, Hua Wang, Xueyan Liu

**Affiliations:** ^1^ Department of Pediatric Neurology Shengjing Hospital of China Medical University Shenyang China

**Keywords:** AP1S2 gene, Chinese pedigree, X‐linked mental retardation

## Abstract

**Background:**

Pettigrew syndrome (PGS) is a rare X‐linked mental retardation that caused by AP1S2 mutation. The pathogenesis of AP1S2 deficiency has remained elusive. The purpose of this study is to give a comprehensive overview of the phenotypic and genetic spectrum of AP1S2 mutations.

**Methods:**

This study systematically analyzed clinical features and genetic information of a Chinese family with AP1S2 variation, and reviewed previously reported literatures with the same gene variation.

**Results:**

We identified a new c.1‐1 G>C mutation in AP1S2 gene from a four generation family with seven affected individuals and found the elevated neuron‐specific enolase (NSE) in a patient. We summarized the clinical manifestation of 59 patients with AP1S2 mutation. We found that pathogenic point mutations affecting AP1S2 are associated with dysmorphic features and neurodevelopmental problems, which included highly variable mental retardation (MR), delayed in walking, abnormal speech, hypotonia, abnormal brain, abnormal behavior including aggressive behavior, ASD, self‐abusive, and abnormal gait. Patients with splice site mutation were more likely to lead to seizures. By contrast, patients with nonsense mutations are more susceptible to microcephaly.

**Conclusion:**

Our findings suggest AP1S2 mutations contribute to a broad spectrum of neurodevelopmental disorders and are important in the etiological spectrum of PGS.

## 1 INTRODUCTION

Intellectual disability (ID), also known as mental retardation (MR) and adaptive behavior, means a person with intellectual and developmental delay result from generalized neurodevelopmental disorder (Piton, Redin, & Mandel, 2013). This disorder can be categorized into two kinds according to its clinical symptoms, radiological or metabolic features: syndromic ID and non‐syndromic ID (Bassani et al., 2013; Tarpey et al., 2009). While cases caused by genetic anomalies have been reported and about 5% of cases are inherited from their parents. It is important to note that X‐linked ID (XLID) is sharp skewed sex ratios: men outnumber women 1.3–1.4 to 1 and XLID is estimated to accounts for up 5%–10% in the males with ID (Gécz, Shoubridge, & Corbett, 2009; Lubs, Stevenson, & Schwartz, 2012). From the identification of the first gene related to XLD to the present, in excess of 100 genes have been found and XLD related gene research has been increasingly valued (Tzschach et al., 2015). The gene number of syndrome was almost equal to the gene number of non‐syndrome (Bassani et al., 2013).

Pettigrew syndrome (PGS) (also called fried‐type syndromic mental retardation) is due to the AP1S2 gene mutation and belongs to the syndrome form of XLD (Cacciagli et al., 2014; Pettigrew, Jackson, & Ledbetter, 1991). PGS are often symptomatic including ID (from moderate to severe) and other highly variable clinical features such as basal ganglia disease, seizures, and Dandy–Walker malformation. In 1972, Fried described a four‐generation family of individuals in nine patients with an X‐linked form of MR (Tarpey et al., 2006). Subsequently, mutations in *AP1S2* gene are gradually discovered and reported (Borck et al., 2008; Cacciagli et al., 2014; Kongsvik, Höning, Bakke, & Rodionov, 2002; Saillour et al., 2007). Current molecular research shows, *AP1S2* gene is located on Xp22.2 and contains five exons. It encoded σ1B subunit of the heterotetrameric adaptor protein‐1 (AP1) and found in the cytosolic side of coated vesicles in the Golgi compartment, thus playing a pivotal role in the recruitment of clathrin and the recognition of sorting signals of transmembrane receptors (Baltes et al., 2014; Glyvuk et al., 2010). Previous studies shown that σ1B which routes for the transport of sortilin exists and is involved in the regulation of adipogenesis and adipose‐tissue mass (Ballarati et al., 2012；Baltes et al., 2014). Until now, probably because of the rarity of PGS, relatively few numbers of aberrance have been reported in *AP1S2,* with almost all of them being nonsense and splice changes scattered throughout the AP1S2 protein. Moreover, the etiologies of PGS are unclear since the first report. The identification of AP1S2 mutation is very important for genetic counseling and prenatal diagnosis.

## 2 MATERIALS AND METHODS

### 2.1 Clinical descriptions

**Figure 1:**
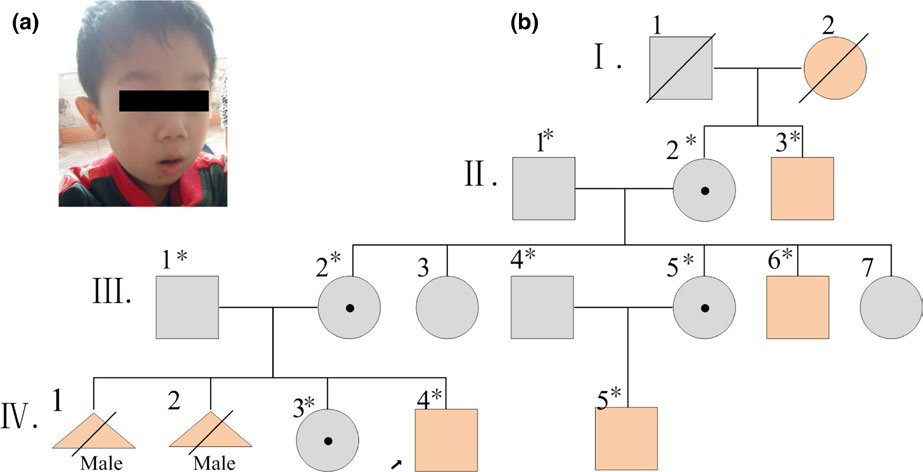
Pedigree of the family. (a) Photograph of the proband at the age of 4 years. (b) Pedigree of the family with deleterious variants in the AP1S2. *Individuals for whom DNA was available

Figure 1b is the pedigree of the patient family. The propositus (patient IV‐4) was born after an uneventful pregnancy (birth weight 3.8 kg). He is the second child (G4P2) to the young, healthy, non‐consanguineous patients. As umbilical cord strangulation, he was full term born by caesarean section. After birth, the patient's growth was normal, but his psychomotor development was delayed. He was able to raise his head at age 8 months and to crawl at age 12 of months.

At 2 years, there was self‐abusive behavior in the form of slapping his head, involuntarily banged his head, episodes of agitation, and temper tantrums which occurs during wakefulness or sleep and head banging was aggravated with common cold. The patient's condition took a favorable turn after physical stimulation. He presented severe development retardation with a developmental quotient (DQ) of 47 (11 months 10 days): movement (10 months), operation (11 months), society (15 months), life (10 months), language (10 months). In the Childhood Autism Rating Scale (CARS), he scored 22 with a total score of less than 30, not consistent with autism spectrum disorder (ASD). The tendon reflexes were normal.

Central nervous system infections and metabolic disease were considered. The blood, urinary and stool routine test, biochemical tests, together with plasma ammonia, plasma lactic acid, creatine kinase isoenzymes, disseminated intravascular coagulation (DIC), blood homocysteine assay, 25‐hydroxyvitamin D3 assay, parathyroid hormone (PTH) was normal. Investigations also included immunoglobulin quantitative determination, cerebral magnetic resonance imaging (MRI), electroencephalogram (EEG), all of which were normal. Routine examination and biochemical analysis of cerebrospinal fluid (CSF) found CSF protein levels were higher than normal. Routine tests of CSF found cerebrospinal fluid was yellowish and transparent, while content of CSF protein (0.69 g/L) was elevated. In serum, neuron‐specific enolase (NSE) was 20.93 ng/ml, while chest radiography and the antibody tests of Chlamydia pneumoniae were normal.

At the age of 2^7^/_12_ years, he was able to speak several single words (i.e., ma, pa, na) and cannot stand alone. After 9 months of rehabilitation training, he walked independently with a broad‐based gait. Subsequently, he could speak fluently. Hypotonia was apparent from 4 years old. A photograph at 4 years showed red thick lower lip, hypertelorism, and posteriorly rotated ears (Figure 1a).

IV‐5, an older cousin of the proband, is a 9‐year‐old boy. He has severe ID. I‐2 died at age 85 years old with moderate ID. II‐3 was born in 1968 and also moderate ID, while III‐6 was 28 years old with mild ID. None of the four patients have abnormal behavior such as aggressive and self‐abusive. Two male fetuses, IV‐1 and IV‐2, were aborted due to hydrocephalus at 36 weeks of gestation and 24 weeks of gestation, respectively.

It is worth noting that all patients had no history of seizures. In addition, none of the obligate carrier females was known to have any symptoms of the disorder and all were of normal intelligence.

### 2.2 Mutation analysis

After getting the informed consent from the family members, blood samples were obtained from three normal males, four affected males, four obligate carriers, and one affected female. To analyze harmful genetic mutations, whole exome sequencing (WES) was performed in the proband (IV‐4). The proband and other family members were confirmed by Sanger sequencing.

WES was performed as previously described. Genomic DNAs were extracted by using the BloodGen Midi Kit (CWBIO, China). Exon‐enriched DNA was captured by Se Ez Exome Enrichment kit V2.0 (47 MB) and sequenced by Illumina HiSeq X10. The sequencing reads were aligned to the human genome (hg19) using BWA. Synonymous changes and SNPs (http://www.ncbi.nlm.nih.gov/projects/SNP) that MAF (minor allele frequency) are higher than 5% were removed. Nonsynonymous changes were filtered using SIFT software (http://sift.jcvi.org).

Four datebase: HGMD (http://www.hgmd.org/), Clinvar (https://www.ncbi.nlm.nih.gov/clinvar/), ESP (http://evs.gs.washington.edu/EVS/), ExAC (http://exac.broadinstitute.org/), and 1000 Genomes (http://www.internationalgenome.org/) were used to search the mutation frequency.

The pathogenicity predictions for the validated splice site mutations were performed using MaxEntScan (MES; http://genes.mit.edu/burgelab/maxent/Xmaxentscan_scoreseq.html), dbscSNV (https://sites.google.com/site/jpopgen/ dbNSFP) and BDGP (BDGP/NNSplice; http://www.fruitfly.org/seq_tools/splice.html) were used.

### 2.3 Review of patients reported in literature

To delineate the phenotypic spectrum associated with *AP1S2* mutations, we collected English articles to search the AP1S2 mutation by using PubMed. The patients for whom no or little clinical information was reported, we listed the phenotype mentioned in the respective publications, but they were not included in summary. Microdeletion involving two or more genes was excluded.

## 3 RESULTS

Using WES, we identified an AP1S2 splice site mutation c.1‐1(IVS1) G>C. The affected males II‐3, III‐6, IV‐4, and IV‐5 were hemizygous. A heterozygous mutation in the same site was observed in the obligate carriers II‐2, III‐2, and III‐5. The unaffected males II‐1, III‐1, and III‐5 were wild‐type allele (Figure 1b). This mutation is consistent with the disease in this family. A search in the HGMD, Clinvar, ESP (http://evs.gs.washington.edu/EVS/), and ExAC, 1000 Genomes confirmed this mutation to be a case first reported in the present study. Hazard prediction of the splice site by Maxentscan and dbscSNV was deleterious (9.37–>1.31). Splice Site Prediction by BDGP indicated that c.1‐1 G>C mutation decreased the strength of the consensus splice‐acceptor site from 90% to 0%. Thus we speculate that this mutation may lead to skipping of exon 2 at the moment of transcription. According to the American College of Medical Genetics and Genomics (ACMG), this variation is pathogenic (PVS1 + PM2 + PP3 + PP1).

**Figure 2:**
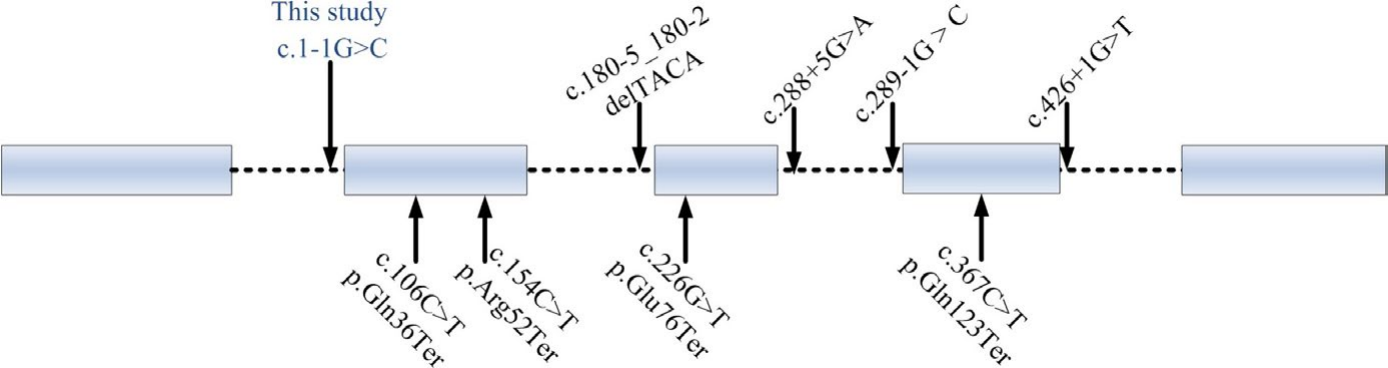
Summary of AP1S2 mutations

Since the AP1S2 gene mutation was identified as the cause of PGS in 2006, only nine AP1S2 mutations have been found in literatures, including eight point mutations and a microdeletion (Bassani et al., 2013; Carpenter, Brown, Qu, & Keenan, 1999; Kongsvik et al., 2002; Pettigrew et al., 1991; Tzschach et al., 2015). Our researches focused on eight mutations in AP1S2. These eight mutations can be of two types: splice site mutations and nonsense mutations (Figure 2). This suggests that both type mutations are highly pathogenic.

Table 1 given an overview of the phenotypic features of patients carrying an AP1S2 mutation. In the two mutation types, the majority of the patient with AP1S2 mutations presented with mental retardation, delayed in walking, hypotonia, coarse facial feature, and abnormal speech. While the features including short stature, aggressive behavior, self‐abusive appeared in some patients. Patients with AP1S2 mutation are described to have some dysmorphic features should be noted: short stature, micropenis, macrocephaly, high forehead, and sagging lips, abnormality of the outer ear (protruding ears and long ears), abnormality of the eye (strabismus and hypermetropia), long nose and face, small pointed jaw, and supernumerary nipple. While microcephaly occur more frequently in nonsense mutations (10/20) than that in the spice site mutations (2/15). On the contrary, the frequency of seizures in the spice site mutations (7/17) was greater than that in the nonsense mutation (1/24). Uncommonly, patients presented with Iron deposition in basal ganglia (IDBG), cerebral calcification, Elevated CSF protein level (ECSFPL), ASD, abnormal gait and vision. Their birth weight and pregnancy were normal.

**Table 1:** Detailed clinical phenotypes of patients with *AP1S2* defects

	Number of patient	Range birth weight in g	Age at death	Dysmorphic features	Microcephaly	Abnormal gait
Nonsense	27	2.440–3.850/*n* = 16	1–73 (mean 23.5)	17/18	10/*n* = 20	3/*n* = 4
Spice site	24	3.000–3.850/*n* = 12	1–73 (mean 22)	12/14	2/*n* = 15	1/*n* = 10
This study	7	3,800/*n* = 1	NA	1/*n* = 1	0/*n* = 7	1/*n* = 1

	Delayed in walking	Abnormal speech^a^	Mental retardation	ASD	Aggressive behavior	Self‐abusive
Nonsense	15/*n* = 16	13/*n* = 15	Mild‐profound	2/2 = 2	10/*n* = 15	5/*n* = 12
Spice site	10/*n* = 11	9/*n* = 11	Borderline‐profound	0/*n* = 1	3/*n* = 8	4/*n* = 15
This study	7/7	7/7	Mild‐severe	1/*n* = 1	0/*n* = 7	1/*n* = 1

	Seizures	Hypotonia	Hydrocephalus	ECSFPL	Cerebral calcification	IDBG
Nonsense	1/*n* = 24	15/15	5/*n* = 24	1/*n* = 1	4/*n* = 4	NA
Spice site	7/*n* = 17	15/15	6/*n* = 18	1/*n* = 1	3/*n* = 3	3/*n* = 5
This study	0/*n* = 7	1/1	2/7	NA	NA	NA

IDBG: iron deposition in basal ganglia; ASD: autism spectrum disorder; ECSFPL; elevated CSF protein level.

a Abnormal speech means speech delay and minimal or absent speech.

## 4 DISCUSSION

PGS, inherited in an X‐linked recessive manner, considered as a rare genetic disease. Up to now, there are only 51 patients with MR have been diagnosed as PGS caused by *AP1S2* mutation. The splice junction c.1‐1 G>C mutation that we identified is most likely to be deleterious for the following reasons: (a) splice site and nonsense mutations in AP1S2 gene have been previously reported in a number of cases and have been shown to cause disease (Figure 2). The mutation site is c.1‐1 G>C in this report, which also belongs to the splice site mutation; (b) the splice site prediction software shown this splice site mutation was deleterious. Meanwhile, the result of BDGP shown this mutation affected the splice site identification; and (c) the splice junction c.1‐3 C>A mutation has proved pathogenic by clinical testing in clinVar. The splice junction c.1‐1 G>C for its location is two bases downstream of the c.1‐3 C>A, which proved c.1‐1 G>C may be harmful. (d) Exon 2 is the translation initiation region in AP1S2 gene. The c.1‐1 G>C splice acceptor site mutation may caused skipping of exon 2, which would then disrupt all amino acids sequence and protein length. (e) Genetically, the mutation of AP1S2 gene and its inheritance are consistent with this disease in this pedigree; (f) According to HVMG criteria, this is also pathogenic. All the above shows the splice site mutation in this study is harmful.

In Tzschach et al. (2015) reported that one out of 150 male patients with intellectual disability had mutations in AP1S2 mutation, so patients with ID should do *AP1S2* detection. However, China has no report on the AP1S2 defect. In past studies, all patients were male and their ages were ranged from 1 to 73 years old (Table 1). Patient I‐1 in this study is the only female patient reported to date. She was 85 years old and is currently reporting the oldest patient.

Although the number of reported subjects with AP1S2‐related MR is so far small, descriptions of these patients are about to reveal a systematic understanding of AP1S2. AP1–σ1B deficient in mice resulted in reduced motor coordination and severely impaired long‐term spatial memory (Baltes et al., 2014). Therefore, the core features consist of highly variable MR in the patients with AP1S2 mutation. At the same time, delayed in walking, abnormal speech, hypotonia, and hydrocephalus were common (Table 1). Some patients could not even talk or walk for their lives (Carpenter et al., 1999; Pettigrew et al., 1991). Nevertheless, in this study, muscular tension was normal before 4 years old, and the occurrence of hypotonia was later than other cases reported. Our review of the literature on AP1S2 mutation turned up abnormal behavior including aggressive behavior, ASD, self‐abusive, and abnormal gait (Kongsvik et al., 2002; Strain, Wright, & Bonthron, 1997; Turner et al., 2003). Even though splice site mutation are more likely to cause seizures, whereas patients with splice site mutation in our study have no seizures features. While the features of self‐abusive, and abnormal gait were found in the patient in this study. Patients with AP1S2 mutation are described to have some dysmorphic features (Table 1). Similarly, patient IV‐4 in this study had mild dysmorphic features. These previous studies, together with our present case series, provide evidence that this feature was considerably influenced by AP1S2 mutation. Although the presence of dysmorphic features in a number of patients described thus far, no specific dysmorphic features has yet been reported. The pedigree reported here had somewhat similar clinical findings with those reported by others, suggesting that AP1S2 contributes significantly to the broad spectrum of dysmorphic features and neurodevelopmental disorders seen in patients with AP1S2 mutation.

In vertebrates, σ1B subunit isoform is highest expression in the brain. In addition, the AP1–σ1B complex deficient mice have damaged synaptic vesicle recycling in hippocampal synapses. The patients that Borck and colleagues described showed the elevated CSF protein and cerebral calcifications can be regarded as diagnostic criteria of PGS (Kongsvik et al., 2002). IDBG also observed in one patient with AP1S2 mutation. Although the patients IV‐4 described in our study also suffer from the elevated of CSF protein, the basal ganglia were normal. In fact, calcium deposits and iron deposition are confusing in PGS (Cacciagli et al., 2014). NSE is found in nerve tissue and neuroendocrine tissue (Rodríguez‐Rodríguez, Egea‐Guerrero, Vilches‐Arenas, Guerrero, & Murillo‐Cabezas, 2014). We observed the elevated of NSE in patient IV‐4 for unknown reason. This study together with the previous reports can speculate that AP1S2 deficiency is associated with neurodevelopmental disabilities that lead to the phenotype observed in our patients.

In conclusion, we present four generations with PGS resulting from a splice site mutation in *AP1S2*. AP1S2 mutations appear to be an important cause of MR. Current knowledge demonstrates that highly variable MR, delayed in walking, abnormal speech, hypotonia, dysmorphic features, abnormal behavior and brain, are suggestive of AP1S2‐related PGS.

## CONFLICT OF INTEREST

The authors declared that they have no conflicts of interest to this work.
